# Knowledge, Attitudes, and Practices of Antibiotic Prescribing Among Arab Dental Students: A Cross-Sectional Study

**DOI:** 10.1016/j.identj.2026.109785

**Published:** 2026-09-04

**Authors:** Mohammad B.A. Sarhan, Mayar Dandneh, Noor R. Al-Hasani, Mohamed Yehia Abdelfattah, Mohammad Mahmoud Hammad, Ashraf AbuKaraky, Zaid B. Al-Bitar, Mohammad H. Al-Shayyab, Ali Ismail Ibrahim, Mariya Salem Ibrahim, Mohammad Hasan Al-Harthy, Deemah A. Almasoud, Antoine Choufani, Khaldoun Darwich, Issam Jamous, Arheiam Arheiam, Fawaz Alzoubi, Latifa Berrezouga, Raghad Saleh, Aya Abu Kwaik, Elham Kateeb

**Affiliations:** a Graduate School of Health Management, Keio University, Fugisawa, Kanagawa, Japan; b Oral Health Research and Promotion Unit, Faculty of Dentistry, Al-Quds University, Jerusalem, Palestine; c College of Dentistry, University of Baghdad, Bab-Almuadham, Baghdad, Iraq; d Oral Biology Department, Faculty of Dentistry, Beni-Suef University, Egypt; e School of Dentistry, The University of Jordan; Faculty of Dentistry, Zarqa University, Jordan; f School of Dentistry, The University of Jordan, Amman, Jordan; g Department of Preventive Dental Sciences, College of Dentistry, Imam Abdulrahman Bin Faisal University, Dammam, Saudi Arabia; h Department of Basic and Clinical Oral Sciences, Faculty of Dental Medicine, Umm Al-Qura University, Makkah, Saudi Arabia; i Division of Dentistry, Faculty of Biology, Medicine, and Health, The University of Manchester, UK; j Pediatric and Public Dental Health Department, Faculty of Dental Medicine, Lebanese University, Beirut, Lebanon; k Faculty of Dental Medicine, Damascus University, Damascus, Syria; l Faculty of Dentistry, University of Benghazi, Benghazi, Libya; m College of Dentistry, Kuwait University, Kuwait; n Faculty of Dental Medicine, University of Monastir, Monastir, Tunisia

**Keywords:** Antibiotic prescribing, Dental students, Antimicrobial resistance, Knowledge, attitudes, and practices, Antibiotic stewardship

## Abstract

**Background:**

Dentists contribute substantially to the global burden of antibiotic use, accounting for approximately 10% of all prescriptions, of which up to 90% might be unnecessary. This exploratory study aimed to assess dental students’ knowledge, attitudes, and antibiotic prescribing practices across ten Arab countries.

**Methods:**

This cross-sectional study used convenience sampling to recruit dental students in clinical years from ten Arab nations: Palestine, Jordan, Syria, Lebanon, Iraq, Egypt, Tunisia, Libya, Kuwait, and Saudi Arabia. From these nations, 2168 dental students completed the survey. Hierarchical multiple regression was used to examine factors associated with inappropriate antibiotic prescribing practices, while linear regression assessed factors associated with attitudes toward appropriate antibiotic prescription.

**Results:**

In Step 1 of the hierarchical regression, demographic variables explained 6.1% of the variance in prescribing practices. Adding access to antibiotic guidance in Step 2 significantly increased the explained variance to 24.5%, while inclusion of actual knowledge, perceived knowledge, and environmental awareness in Step 3 further increased the explained variance to 33.2%. In the final step, adding attitude and confidence scales resulted in a small additional contribution, explaining 33.7% of the variance. Being a female student, an intern, and coming from a middle-income country were associated with less inappropriate antibiotic prescribing. Perceived knowledge (β = −0.392) had a stronger association with appropriate prescribing than actual knowledge (β = −0.092). More favourable attitudes toward antibiotic use were significantly associated with reduced inappropriate prescribing (β = −0.149).

**Conclusions:**

Appropriate antibiotic prescribing among dental students was associated with demographic characteristics, access to antibiotic guidance, knowledge, and attitudes toward antibiotic use. Perceived knowledge showed a stronger association with prescribing practices than actual knowledge, while favourable attitudes toward antibiotic stewardship were associated with less inappropriate prescribing. These findings suggest that educational interventions should extend beyond factual knowledge to strengthen self-efficacy and stewardship.

## Introduction

Bacterial antimicrobial resistance (AMR) occurs when bacteria no longer respond to antimicrobial agents that were previously effective against them, making infections more difficult to treat and increasing the risk of disease transmission, severe illness, and death. As a result, AMR has emerged as one of the greatest threats to global health and development in the 21st century.^1^^,^^2^ It is estimated that AMR is one of the leading causes of death globally, being associated with an estimated 4·71 million deaths and directly causing 1.14 million deaths in 2021,^3^^,^^4^ and these numbers are projected to reach 10 million annual deaths by 2050.^5^ Although the death rates of AMR decreased among children under five by 50% between 1990 and 2021, they increased by 80% among those older than 70 years old.^4^ This indicates that a huge burden on both clinical and public health aspects is associated with AMR, and this burden is increasing over time.^6^

The World Health Organization (WHO) reported in its new Global Antibiotic Resistance Surveillance Report 2025 that, in the year 2023, antibiotic-resistant bacteria caused one-sixth of all the globally confirmed bacterial infections.^3^ Although this is a global issue, its prevalence varies among regions. AMR was mostly found in one-third of the laboratory-confirmed bacterial infections in the Southeast Asia and Eastern Mediterranean regions, compared to 1 in 5 and 1 in 10 cases in African and European regions, respectively.^3^ Ultimately, this global health issue has led to an increased prevalence of untreatable infections, unsafe routine medical procedures, prolonged hospital stays, and high healthcare costs,^3^^,^^7^ thereby undermining the progress made in the field of medicine over recent decades.^3^

The prevalence of inappropriate antibiotic prescription varied widely in the literature. A systematic review targeted low- and middle-income countries reported that this prevalence ranged between 7.9% and 100%.^7^ In primary health care, a review reported a pooled estimate of 57.6% of antibiotic prescribing was inappropriate.^8^ Another review reported that the global average of inappropriate antibiotic prescription was 37%, which was estimated based on inappropriate dosage, duration, or choice of antibiotics.^9^ Therefore, inappropriate antibiotic use – excessive and unnecessary prescription – is a major contributor that drives the development of AMR.^5^^,^^10^

Health professionals, including dentists, play a critical role in ensuring the appropriate prescribing and administration of antibiotics, as well as educating patients about their responsible use.^10^ In dentistry, antibiotics are primarily prescribed for therapeutic and prophylactic purposes.^11^^,^^12^ Their use in managing orofacial and odontogenic infections has become a central component of general dental practice.^11^ Additionally, antibiotics are prescribed for prophylactic purposes, particularly for certain immunocompromised patients and for the prevention of infective endocarditis (IE). Although recommendations regarding antibiotic prophylaxis for IE vary across international guidelines, most contemporary guidelines advocate a restrictive approach, limiting prophylactic antibiotic use to patients at highest risk of adverse outcomes undergoing specific invasive dental procedures.^13^

Despite these limited indications, dentists widely use antibiotics for therapeutic purposes, often beyond recommended guidelines. For example, a study in Wales found that 70.6% of prescribed antibiotics were issued without any accompanying clinical procedure.^14^ This finding highlights a concerning trend: antibiotics are sometimes used as substitutes for definitive local operative treatments, such as tooth extraction, endodontic therapy, or incision and drainage, which remain the cornerstone of managing acute odontogenic infections.^14^

Globally, evidence suggests that dentists play a notable role in the escalating public health crisis of antibiotic resistance.^10^^,^^15^^,^^16^ Dentists in primary care are estimated to account for approximately one-tenth of all antibiotic prescriptions,^17^ which is double the rate of emergency medical services.^18^ Similar patterns have been reported internationally. In the United Kingdom, dental professionals were responsible for 11% of total antibiotic prescriptions – equivalent to 67 out of every 616 prescriptions per 1000 people.^18^ The proportion of antibiotics prescribed by dentists varies by country: 3% in Australia,^19^ 5.8% in Belgium,^13^ 13.9% in Germany,^20^ and 15.6% in Norway.^21^

Alarmingly, a substantial proportion of these prescriptions appear to be unnecessary or inappropriate, further intensifying the problem of antimicrobial resistance.^10^ For instance, a study in Spain revealed that half of the surveyed dentists prescribed antibiotics inappropriately in nearly 29% of clinical cases.^22^ Similarly, in Wales, 57.4% of patients received antibiotics, and 65.6% of those prescriptions were issued without evidence of spreading infection.^14^ Such data underscore the pressing need for stronger antibiotic stewardship in dental practice to mitigate misuse and its global consequences. This proportion of antibiotic prescribing practice among dentists that is not compatible with guidelines is significantly high, reaching up to 90% in some high-, middle-, and low‐income countries such as the UK, Lebanon, and Ghana.^19^

The problem of antibiotic overuse in dentistry is not confined to Western countries; it is also a growing concern across the Arab region. In Jordan, for instance, a survey of 204 dentists revealed that only 9.4% were aware of the existing national guidelines and action plans on antimicrobial resistance (AMR).^23^

The challenge of promoting appropriate antibiotic prescribing in dentistry is also evident across the Arab region.^24^, ^25^, ^26^, ^27^ In Lebanon, an evaluation of antibiotic prescribing among dentists in Beirut found that although antibiotics were frequently prescribed for prophylactic and therapeutic purposes, their appropriateness was low. Only 1%, 6.7%, and 11.1% of prescriptions for implant surgery, tooth extraction, and endodontic treatment, respectively, were considered appropriate. Similarly, only 1.7% of prescriptions for periodontal abscesses and 1.9% of those prescribed for pulpal diseases and periradicular complications were judged appropriate. Even when the selected antibiotic complied with guidelines, the prescribed dosage and duration were inappropriate in most prophylactic cases.^25^ In Saudi Arabia, inappropriate and unnecessary antibiotic prescriptions in healthcare settings, including dentistry, have been reported to range from 24% to 80%.^26^ Likewise, among dentists in Muscat, Oman, 69.1% of antibiotic prescriptions were found to be inappropriate.^28^ These findings underscore the need to strengthen antibiotic stewardship and adherence to evidence-based prescribing practices in the region.

Such findings reflect a pattern of frequent and often unjustified antibiotic use across the region, which aligns with broader reports of an increasing incidence of antibiotic-resistant nosocomial infections in Arab countries.^29^ In the past 20 years, resistance rates to various antimicrobial agents significantly increased; for example, resistance to cephalosporin antibiotics increased from 37% to 89.5%.^30^ Consequently, these patterns underscore the alarming escalation of the antimicrobial resistance (AMR) burden in the region and highlight the urgent need for effective stewardship policies, continuing education, and adherence to evidence-based prescribing guidelines.^29^^,^^31^

Despite the growing burden of antimicrobial resistance, research on antibiotic prescribing practices among dentists in the Arab region remains fragmented and limited in scope. Most existing studies are country-specific, such as those conducted in Jordan,^23^^,^^32^ Lebanon,^24^ the United Arab Emirates,^25^ and Saudi Arabia.^27^^,^^33^ Collectively, these investigations represent isolated efforts rather than a coordinated or comprehensive research agenda.

Although interventions are ultimately planned and implemented at the national level, the drivers of inappropriate antibiotic use often transcend national boundaries. These drivers include clinician-related factors (eg, Workload, competing demands, habits, educational gaps, skills, and clinical uncertainty), patient-related factors (eg, relationships, expectations, and satisfaction), sociopolitical factors (eg, healthcare context, access to the right care, and incentives for and against antibiotic use), and clinical context-related factors (eg, Influence of peers and colleagues, and other practice characteristics such as insurance provision or geographic location).^34^^,^^35^ A multicountry perspective, therefore, enables the identification of shared regional patterns, highlights context-specific variations, and provides a comparative framework for designing contextually informed yet regionally harmonized policies and educational interventions.^36^, ^37^, ^38^ Such evidence is particularly critical for the Arab region, where countries share similar health system structures, educational models, and sociocultural factors that influence clinical decision-making.

Dental students are future prescribers, and educational gaps may translate into inappropriate prescribing behaviours later in professional practice. Therefore, understanding dental students’ knowledge, attitudes, and prescribing practices is essential for designing effective educational interventions. However, there is a notable paucity of research focusing on future dental practitioners, who will play a pivotal role in shaping effective antibiotic stewardship practices. Existing studies on dental students’ prescribing behaviours have been conducted predominantly in Saudi Arabia,^39^, ^40^, ^41^, ^42^ with only one recent study involving Palestinian students.^43^ This limited and fragmented evidence base highlights the need for broader, comparative research to understand how educational systems, clinical training environments, and professional norms across countries influence prescribing behaviour.

Accordingly, this study aims to address these gaps by conducting an exploratory, multicountry assessment of dental students’ knowledge, attitudes, and antibiotic-prescribing practices across 10 Arab countries. Specifically, the study sought to examine the levels of knowledge, attitudes, and prescribing practices related to antibiotic use among Arab dental students and to investigate whether knowledge attitudes associated with prescribing practices. We hypothesized that students with higher levels of knowledge and more favourable attitudes toward antibiotic stewardship would demonstrate more appropriate antibiotic prescribing practices. By adopting a regional perspective, this work seeks to generate evidence to inform national education programs while fostering region-wide strategies to strengthen antibiotic stewardship and combat the growing threat of antimicrobial resistance in the Arab world.

## Methods

### Study design and data collection

This exploratory cross-sectional study employed a convenience sampling approach targeting dental students in clinical years (4th, 5th, and 6th [internship year]) from ten Arab countries: Palestine, Jordan, Syria, Lebanon, Iraq, Egypt, Tunisia, Libya, Kuwait, and Saudi Arabia. As participation was based on voluntary response to an online survey, the findings should be interpreted with consideration of the potential limitations in representativeness inherent to convenience sampling. Data were collected using a self-administered questionnaire hosted on Google Forms. The survey link was distributed through faculty collaborators at dental schools across the participating countries using institutional emails and professional social media platforms (eg, Facebook). To enhance participation rates, three reminder messages were circulated following the initial invitation during the data collection period, which spanned from September 2023 to February 2024.

### Study variables

The items of the questionnaire used to measure the outcome and independent variables were adapted from a Jordanian study that assessed healthcare professionals’ knowledge, attitudes, and behaviours regarding antibiotic prescription and antibiotic resistance.^44^ The complete scales used in this study are available in Supplement 1. The Theory of Planned Behaviour (TPB) was used as a conceptual framework to interpret the relationships among study variables, mainly knowledge, attitudes, and antibiotic prescribing practices.^45^

### Antibiotic prescription practices

The main outcome variable of this study is the appropriate use of antibiotic prescription practices. It was measured using an 11-item scale. Nine items assessed inappropriate practices, while two assessed good or proper practices. The items were evaluated using a 6-item Likert scale; ‘Once a day’ = 1, ‘More than once a day’ = 2, ‘Once a week’ = 3, ‘More than once a week’ = 4, ‘Rarely’=), ‘Never’ = 6. The total score of the scale ranged from 11 to 66, with lower scores indicating better practice and higher scores indicating inappropriate practices. Cronbach’s alpha for the scale was 0.827.

### Actual and perceived knowledge

The students answered seven statements to assess their actual knowledge of antibiotic use and resistance. Examples of these statements include: ‘Unnecessary use of antibiotics makes them ineffective’ and ‘Antibiotic-resistant bacteria can spread from person to person’. The participants had to choose if each statement was ‘True’ or ‘False’ with an additional option of ‘Unsure’. The total score, which ranges from 0 to 7, was measured by counting the number of correct answers. On the other hand, assessing perceived knowledge was based on students’ responses to a three-item scale. An example of the items includes: ‘I know what antibiotic resistance is’. Cronbach’s alpha of the perceived knowledge scale was 0.869, with a total score that ranges from 3 to 15. A higher score indicates a higher level of perceived knowledge.

### Attitude

The attitude scale consisted of two items: ‘I have a key role in helping control antibiotic resistance’, and ‘There is a connection between my prescribing practices and antibiotic resistance’. The attitude scale had a Cronbach’s Alpha of 0.752. The total score ranges from 2 to 10, with higher scores indicating a better attitude towards antibiotic use.

### Antibiotic prescription guidance accessibility (opportunity)

This scale assesses how students perceive their opportunities to access guidelines that enable them to use and prescribe antibiotics properly. An example of one item on this scale is: ‘I have easy access to guidelines I need on managing infections’. The minimum possible score was 3, while the maximum was 15. Higher scores indicated better perceived opportunities. Cronbach’s alpha for this scale was 0.867.

### Environmental awareness

Environmental awareness was assessed using two statements that link environmental factors, such as water waste and excessive antibiotic use in livestock, to antibiotic resistance. Cronbach’s alpha of this scale was 0.761. A higher score on the environmental factors scale indicates a greater awareness of environmental factors, with a possible score range of 2 to 10.

### Confidence

This scale measures students’ confidence in the available guidelines and in their ability to make antibiotic prescribing decisions. A total score ranges from 2 to 10, with a Cronbach’s alpha of 0.866.

### Statistical analysis

Data were collected from dental students in their clinical years across ten Arab countries. Sampling was convenience-based in all participating countries except Palestine, where a representative and adequately powered national sample was achieved. Because samples from most countries were not representative, all primary analyses were conducted on the pooled sample. Country-level comparisons were presented descriptively and interpreted with caution, as they reflect the characteristics of participating students rather than national populations.

We reported frequencies for categorical variables, while means and standard deviations were reported for continuous variables. The countries of the universities were classified according to the World Bank (WB) income classification for 2026:^46^ (1) High income (Kuwait and Saudi Arabia), (2) Upper-middle income (Iraq and Libya), (3) Low-middle income (Palestine, Jordan, Tunisia, Lebanon, and Egypt), and (4) Low income (Syria).

Correlation coefficients were calculated between the scales of actual knowledge, perceived knowledge, attitudes, practice, confidence, opportunity, and awareness of environmental factors. We conducted a hierarchical multiple regression analysis to explore the factors associated with inappropriate antibiotic prescribing practices among university students. Lower scores in the practice scale indicate appropriate prescribing practices, while higher scores indicate inappropriate ones. Independent variables were entered in four sequential steps, as explained in Table 1.

**Table 1:** Description of factors included in the hierarchical multiple regression.

Step	Predictors included	Notes
Step 1	Country (ref: Egypt), Gender (ref: male), No family medicine training, year (ref: interns)	Demographics
Step 2	Opportunity	Access to materials/guidelines
Step 3	Knowledge, Perceived Knowledge, Environmental awareness	All reflect factual/belief-based awareness
Step 4	Attitude & Confidence	Value-based, motivational constructs

Categorical variables were dummy-coded. The dummy variable for the reference group was not included in the regression models.

The change in explained variance (ΔR²) and the statistical significance of factors were reported. Multicollinearity was examined by checking the variance inflation factors (VIF). VIF values lower than 10 indicate no multicollinearity. All analyses were conducted using IBM SPSS Statistics (version 30) and JASP (Version 0.19.3). A *P*-value of < .05 was considered statistically significant. Finally, a linear regression model was conducted to assess the factors associated with students’ attitudes towards proper antibiotic prescription.

## Results

### Demographics

Among the 2168 dental students and interns who completed the survey from ten Arab countries, 59.5% were women. The percentage of females varied by country, with 81.8% of the Tunisian students being women, followed by 75.6% of Libyan students, and 71.0% of Jordanian students. On the other hand, Saudi male students (57.7%) were more than female ones. The proportion of interns was highest in Tunisia (72.7%) and Saudi Arabia (60.4%). Around 30% of the participants were interns, while those in the 4th and 5th years were 34.2% and 36.3% of the study population, respectively. Finally, 60.3% of dental students have a relative who works in the medical field, with Kuwait (77.8%) and Palestine (59.5%) having the highest rate among other countries. For further details, refer to Table 2 and Supplement 2.

**Table 2:** Demographic characteristics.

Variable	Category	Total *N* (%)
Sex	Female	1291 (59.5)
	Male	877 (40.5)
Year of study	4th Year	741 (34.2)
	5th Year	786 (36.3)
	Interns	641 (29.6)
Do you have a family member/relative working in the medical field?	No	861 (39.7)
	Yes	1307 (60.3)
Total *N* = 2168		

### KAP scales

As shown in Table 3, the mean score for actual knowledge was 4.6 (SD 1.5) out of 7, and the perceived knowledge score was 10.2 (SD 3.4) out of 15, while the attitudes scale’s mean score was 6.7 (SD 2.3) out of 10. The overall practice mean score was 34.1 (SD 12.1). Other scales, such as environmental awareness, confidence, and access to antibiotic prescription guidance, had mean scores of 6.3 (SD 2.2) out of 10, 6.4 (SD 2.3) out of 10, and 9.3 (SD 3.2) out of 15, respectively.

**Table 3:** Means and standard deviations for the KAP and other scales.

Variable	Egypt	Iraq	Jordan	Kuwait	Lebanon	Libya	Palestine	Saudi Arabia	Syria	Tunisia	Total Sample
**Knowledge**	4.7 (1.4)	4.6 (1.6)	5.1 (1.3)	4.3 (1.5)	5.3 (1.3)	4.8 (1.4)	4.2 (1.6)	4.8 (1.6)	4.7 (1.4)	4.3 (1.3)	**4.6 (1.5)**
**Perceived Knowledge**	9.9 (3.6)	10.7 (3.0)	10.7 (3.4)	9.6 (4.3)	11.5 (3.3)	8.8 (3.8)	9.5 (3.4)	11.2 (3.6)	10.9 (3.1)	11.1 (2.6)	**10.2 (3.4)**
**Attitude**	6.5 (2.4)	6.9 (2.0)	7.2 (2.6)	6.6 (2.7)	7.5 (2.2)	5.6 (2.4)	6.4 (2.3)	7.1 (2.6)	7.1 (1.9)	7.0 (2.1)	**6.7 (2.3)**
**Overall Practice**	37.7 (12.3)	31 (11.6)	28.8 (11.6)	34.2 (11.7)	35.1 (12.3)	40.2 (11.2)	37.5 (11.1)	28.8 (11.7)	30.5 (11.2)	30 (12.7)	**34.1 (12.2)**
**Environ-mental awareness**	6.0 (2.3)	6.5 (2.1)	6.5 (2.2)	6.2 (2.6)	6.9 (2.2)	5.4 (2.0)	6.1 (2.3)	6.6 (2.5)	6.1 (1.9)	7.0 (1.8)	**6.3 (2.2)**
**Confidence**	6.0 (2.2)	6.7 (2.1)	6.3 (2.4)	5.8 (2.7)	7.0 (2.1)	5.5 (2.2)	6.1 (2.3)	6.9 (2.7)	6.5 (2.1)	7.1 (1.8)	**6.4 (2.3)**
**Opportunity**	8.8 (3.3)	9.5 (2.8)	9.0 (3.2)	9.5 (4.0)	10.5 (3.0)	8.0 (2.9)	9.0 (3.2)	10.2 (3.8)	9.1 (2.7)	9.6 (2.9)	**9.3 (3.2)**

### Correlations

**Fig. 1:**
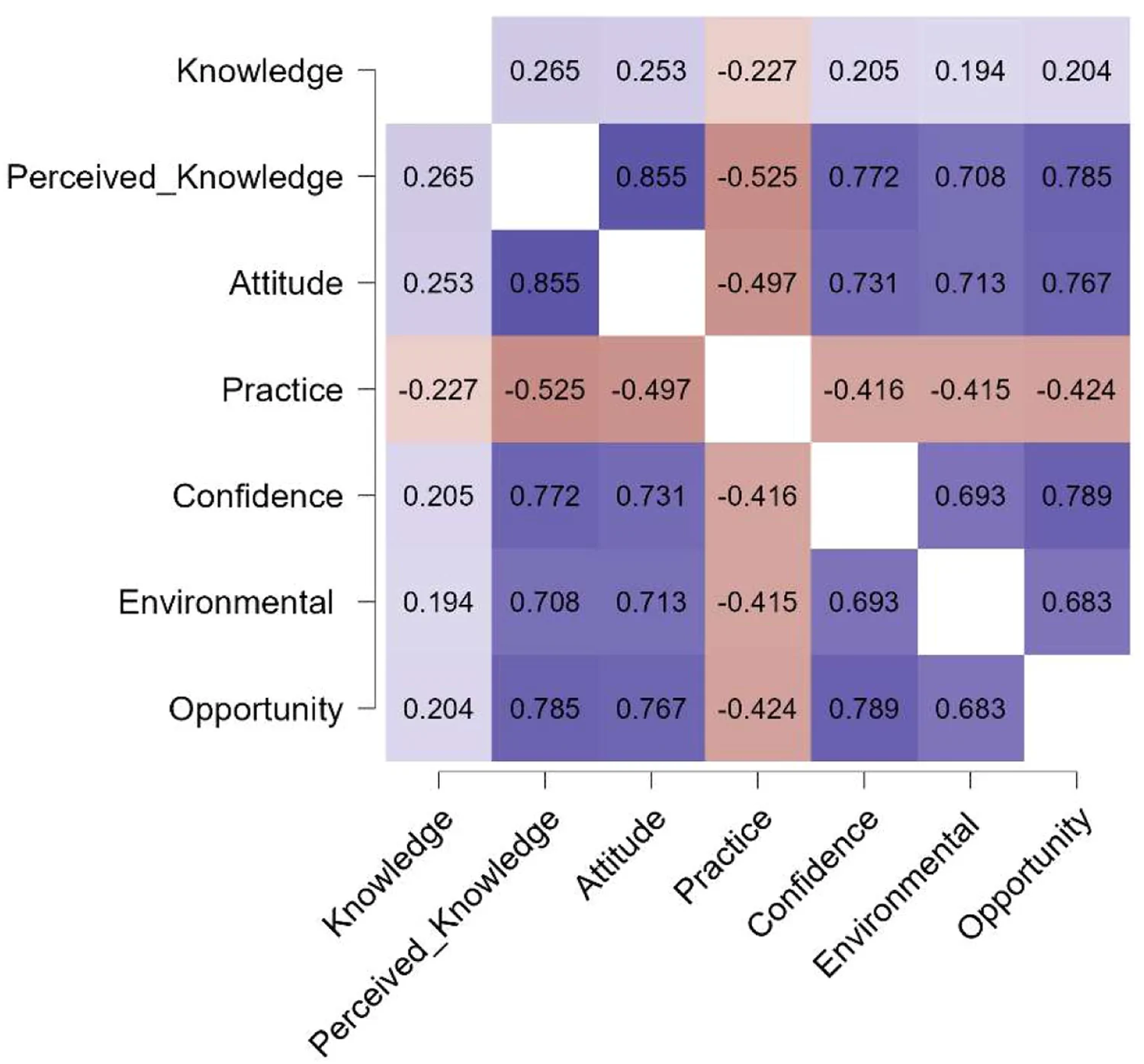
Heat map of the correlations among KAP and other scales.

As shown in Table 4 and Figure 1, correlation analysis revealed that actual knowledge exhibited significant weak positive correlations with perceived knowledge, attitude, confidence, environmental awareness, and opportunity, with correlation coefficients ranging from 0.194 to 0.265. On the other hand, it exhibits a weak negative correlation with overall practice (*r* = −0.227). Perceived knowledge had a very strong positive correlation with attitude (*r* = 0.855), confidence (*r* = 0.772), environmental awareness (*r* = 0.708), and opportunity (*r* = 0.785), and a moderate negative correlation with practice (*r* = −0.525). Attitude had a moderate negative correlation with practice (*r* = −0.497). Practice showed moderate negative correlations with confidence (*r* = −0.416), environmental awareness (*r* = −0.415), and opportunity (*r* = −0.424). Finally, confidence, environmental awareness, and opportunity were strongly positively correlated with each other, with coefficients ranging from 0.683 to 0.789. All correlations were statistically significant (*P* < .05).

**Table 4:** Pearson’s correlations among KAP and other scales.

Variable		Knowledge	Perceived Knowledge	Attitude	Practice	Confidence	Environmental	Opportunity
Knowledge	*n*	–						
	*r*	–						
	*P*	–						
Perceived Knowledge	*n*	2048	–					
	*r*	0.265	–					
	*P*	<.001	–					
Attitude	*n*	2056	1979	–				
	*r*	0.253	0.855	–				
	*P*	<.001	<.001	–				
Practice	*n*	1412	1371	1363	–			
	*r*	−0.227	−0.525	−0.497	–			
	*P*	<.001	<.001	<.001	–			
Confidence	*n*	2058	1968	1974	1363	–		
	*r*	0.205	0.772	0.731	−0.416	–		
	*P*	<.001	<.001	<.001	<.001	–		
Environmental	*n*	1894	1819	1823	1278	1833	–	
	*r*	0.194	0.708	0.713	−0.415	0.693	–	
	*P*	<.001	<.001	<.001	<.001	<.001	–	
Opportunity	*n*	1968	1904	1911	1322	1916	1779	–
	*r*	0.204	0.785	0.767	−0.424	0.789	0.683	–
	*P*	<.001	<.001	<.001	<.001	<.001	<.001	–

*P, P*-value; *r*, Pearson’s *r*.

### Hierarchical multiple regression

In Step 1, demographic variables accounted for 6.1% of the variance in the overall practice (R² = 0.061, *P* < .001). In Step 2, the addition of the antibiotic guidance accessibility had a significant impact in improving the model with ΔR² = 0.184, bringing the total explained variance to 24.5%. Introducing actual and perceived knowledge, along with the environmental awareness scale, significantly increased the total explained variance in Step 3 (ΔR² = 0.087, R² = 0.332, *P* < .001). In the final step, adding attitude and confidence scales resulted in ΔR² = 0.005. Despite being small, this change in R² was statistically significant, with a final R² = 0.337.

As shown in Table 5, being a female student (β = −0.084, *P* < .001), an intern (β = 0.094, *P* = .001), and having better actual (β = −0.092, *P* < .001), perceived knowledge (β = −0.392, *P* < .001), and coming from a middle income country (Upper:β = 0.098, Lower: β = 0.202) were associated with less inappropriate antibiotic prescribing. More favourable attitudes were associated with less inappropriate antibiotic prescribing (β = −0.149, *P* = .007).

**Table 5:** Hierarchical multiple regression^‡^ showing the factors associated with overall practice.

Predictors	Step 1 β† (*P*-value)*	Step 2 β† (*P*-value)*	Step 3 β† (*P*-value)*	Step 4 β† (*P*-value)*
Female	−0.093 (.001)	−0.099 (<.001)	−0.086 (<.001)	−0.084 (<.001)
Fifth year	0.049 (.135)	0.033 (.263)	0.027 (.336)	0.026 (.35)
Interns	0.087 (.011)	0.094 (.002)	0.094 (.001)	0.094 (.001)
No family in the medical field	0.032 (.262)	0.036 (.157)	0.035 (.144)	0.033 (.172)
WB income: Upper-middle	0.129 (.007)	0.076 (.079)	0.100 (.013)	0.098 (.015)
WB income: Lower-middle	0.306 (<.001)	0.215 (<.001)	0.198 (<.001)	0.202 (<.001)
WB income: Low	0.003 (.916)	−0.024 (.414)	−0.011 (.685)	−0.007 (.802)
Opportunity	–	−0.434 (<.001)	−0.004 (.922)	0.013 (.799)
Actual knowledge	–	–	−0.097 (<.001)	−0.092 (<.001)
Perceived knowledge	–	–	−0.474 (<.001)	−0.392 (<.001)
Environmental awareness	–	–	−0.019 (.635)	0.0001 (.997)
Confidence	–	–	–	0.024 (.638)
Attitude	–	–	–	−0.149 (.007)
**R²**	**0.061**	**0.245**	**0.332**	**0.337**
**Adjusted R²**	**0.055**	**0.240**	**0.326**	**0.329**
**ΔR²**	–	**0.184**	**0.087**	**0.005**

Note: Dependent variable: Higher scores indicate more inappropriate antibiotic prescribing behaviour.

⁎ *P* < .05, which is considered statistically significant.

† Standardized regression coefficients (β) are reported.

The linear regression results showed that actual knowledge (β = 0.032, *P* = .015), perceived knowledge (β = 0.569, *P* < .001), confidence (β = 0.058, *P* = .028), and practice (β = −0.041, *P* = .007) were significantly associated with the ‘attitudes’ total score. Further details are shown in Table 6.

**Table 6:** Linear regression analysis of factors associated with the attitude total score.

Predictor	Standardized β (*P*-value)
Female	0.008 (.519)
Fifth year	−0.001 (.971)
Interns	0.010 (.518)
No family medicine training	−0.010 (.431)
Upper-middle income country (WB)	−0.006 (.780)
Lower-middle income country (WB)	0.036 (.100)
Low-income country (WB)	0.028 (.056)
Opportunity	0.160 (<.001)
Knowledge	0.032 (.015)
Perceived knowledge	0.569 (<.001)
Environmental factors	0.141 (<.001)
Confidence	0.058 (.028)
Practice	−0.041 (.007)

## Discussion

This study assessed the factors associated with inappropriate prescription of antibiotics among dental students in ten Arab countries. Limited actual knowledge was one of the factors associated with inappropriate prescribing. However, it turned out that perceived knowledge and positive attitudes showed a stronger association with better prescribing practices and behaviours.

Given that the mean score of the actual knowledge was moderate, the results show that actual knowledge was weakly correlated with perceived knowledge. Although this might appear counterintuitive, health and education research have revealed that these constructs are distinct, and using them interchangeably is not recommended.^47^ A systematic review reported that physicians’ knowledge and perceptions may differ in low- and middle-income countries (LMICs). For example, they might agree on the importance of reducing antibiotic use, but still believe that prescribing narrow-spectrum antibiotics is safe.^48^ In China, one study reported that 92% of medical students agreed that inappropriate use of antimicrobials can be harmful for patients; however, their average score on knowledge questions was 3.78 out of 11, indicating poor factual knowledge.^49^ One study in Belgium found that dental practitioners’ knowledge levels differed from their satisfaction with information on antibiotics, despite having high overall satisfaction with the information.^50^

Furthermore, the weak correlation between actual and perceived knowledge might suggest that students do not have the ability to accurately evaluate their own knowledge and skills of prescribing antibiotics. In other words, students might either overestimate or underestimate their prescribing skills and competencies. Eventually, inaccurate self-assessment can hinder appropriate prescribing practices. To address this discrepancy between actual and perceived knowledge, there is a need for an educational approach that amalgamates knowledge-gaining with regular assessment and feedback, followed by self-reflection and adjustment of practices.^51^

Actions are needed to address the lack of congruence between actual and perceived knowledge of antibiotic treatment guidelines.^52^ Continuing education is one approach that can help reduce the misalignment between the actual and perceived knowledge.^53^ Furthermore, local authorities need to provide clear guidelines for antibiotic prescription that are easily accessible to all dentists. This should be followed by ensuring that dentists implement and use these guidelines.^52^ Encouraging dentists’ commitment to the guidelines can be achieved through educational interventions. In a Norwegian educational intervention, general practitioners were informed about the national antibiotic use guidelines, best practice for treating acute respiratory tract infections, and feedback on their previous year’s antibiotic prescribing profile. The results of this intervention led to a 28% reduction in the odds of prescribing unnecessary antibiotics while treating respiratory tract infections.^54^ Audit-based strategies were found to be effective in achieving proper antibiotic stewardship among dentists, especially when combined with education, feedback, and behaviour change messages tailored to the particular attitudes of each dentist.^55^, ^56^, ^57^ Through this approach, dentists can use the received feedback or education for self-reflection on their prescribing practices in order to adjust them according to evidence-based guidelines.^57^

Furthermore, the results showed a weak correlation between the actual knowledge and confidence scale among dental students in our study. Similarly, a study conducted at a US university teaching hospital reported no difference in the level of actual knowledge between students who reported confidence in their antimicrobial use and those who were less confident.^58^ In DR Congo, medical doctors and students had high confidence in their knowledge; however, this confidence was not associated with the factual knowledge score.^59^ Similarly, dental students from Jordan, Australia, Sri Lanka, Japan, and Vietnam reported proper awareness of the antibiotic resistance process, but this knowledge was not transformed into confident prescribing practices.^44^^,^^60^

Contrary to actual knowledge, the results showed a strong correlation between perceived knowledge and the confidence scale among dental students in our study. While both constructs are close, perceived knowledge can reflect a person’s subjective belief of having an understanding of a specific topic, not their own ability to perform a specific task.^61^ The subjective perception of low knowledge can be a motivator for practitioners to seek information from various sources to address their knowledge gaps.^61^ Several studies have reported that junior doctors may have a lower level of perceived knowledge, leading them to consult with their seniors and colleagues.^48^^,^^50^ Mainjot et al^50^ emphasized that this indicates poor efficacy and validity of official information sources (eg, university continuing education). On the other hand, acknowledging self-weaknesses and then seeking help to address this gap could lead practitioners (ie, dentists) to feel more confident in their treatment-related decisions.^48^

However, perceived knowledge was associated with lower rates of inappropriate antibiotic prescribing among dental students. A better-perceived knowledge score had a stronger association with adequate prescribing practices. In Scotland, junior doctors perceived that the lack of knowledge could lead to errors in their antibiotic prescribing practices.^62^ In contrast to our results, a French study found that the majority of dentists they surveyed had inappropriate antibiotic prescription practices. The majority underestimated their lack of knowledge, as around 60% felt they were properly informed or trained on how to use antibiotics.^63^ Similarly, in Belgium, there was incoherence between practitioners’ high satisfaction with their knowledge and their prescription behaviours, such as endocarditis or artificial joint infections prophylaxis, which were not compatible with the international guidelines.^50^

Regardless of its high correlation with perceived knowledge, confidence was not a significant predictor of appropriate prescribing practices in this study in the hierarchical regression. Similar results were found among dental students in Chicago enrolled in the University of Illinois Chicago. Their confidence in antibiotic prescribing decision-making did not determine their likelihood of prescribing antibiotics.^64^ Furthermore, in a study targeting dental students of six universities in Norway, Canada, and Brazil, students showed a good level of awareness about the relevance of antibiotic resistance in dental practice. Those with high awareness had the highest confidence in their antibiotic prescription abilities. However, almost 10% reported strong confidence in selecting the most appropriate antibiotic regimen for treating infections, and only a quarter of them were very confident in communicating with patients when antibiotics are not needed.^65^ This suggests that having confidence in the knowledge gained – although correlated with practice – does not necessarily translate into better antibiotic use practices. This can align with what was found in Nepal, where Nepalese dental students who lacked knowledge and confidence had a lower ability to identify cardiac conditions, and consequently, overprescribed antibiotic prophylaxis.^66^ Based on the previous evidence, the mismatch between confidence in prescribing abilities and actual practices implies that factors other than self-confidence may be associated with appropriate prescribing practices. In our study, students’ attitudes toward antibiotic prescribing were associated with appropriate prescribing practices, indicating that they may play a role alongside other factors.

**Fig. 2:**
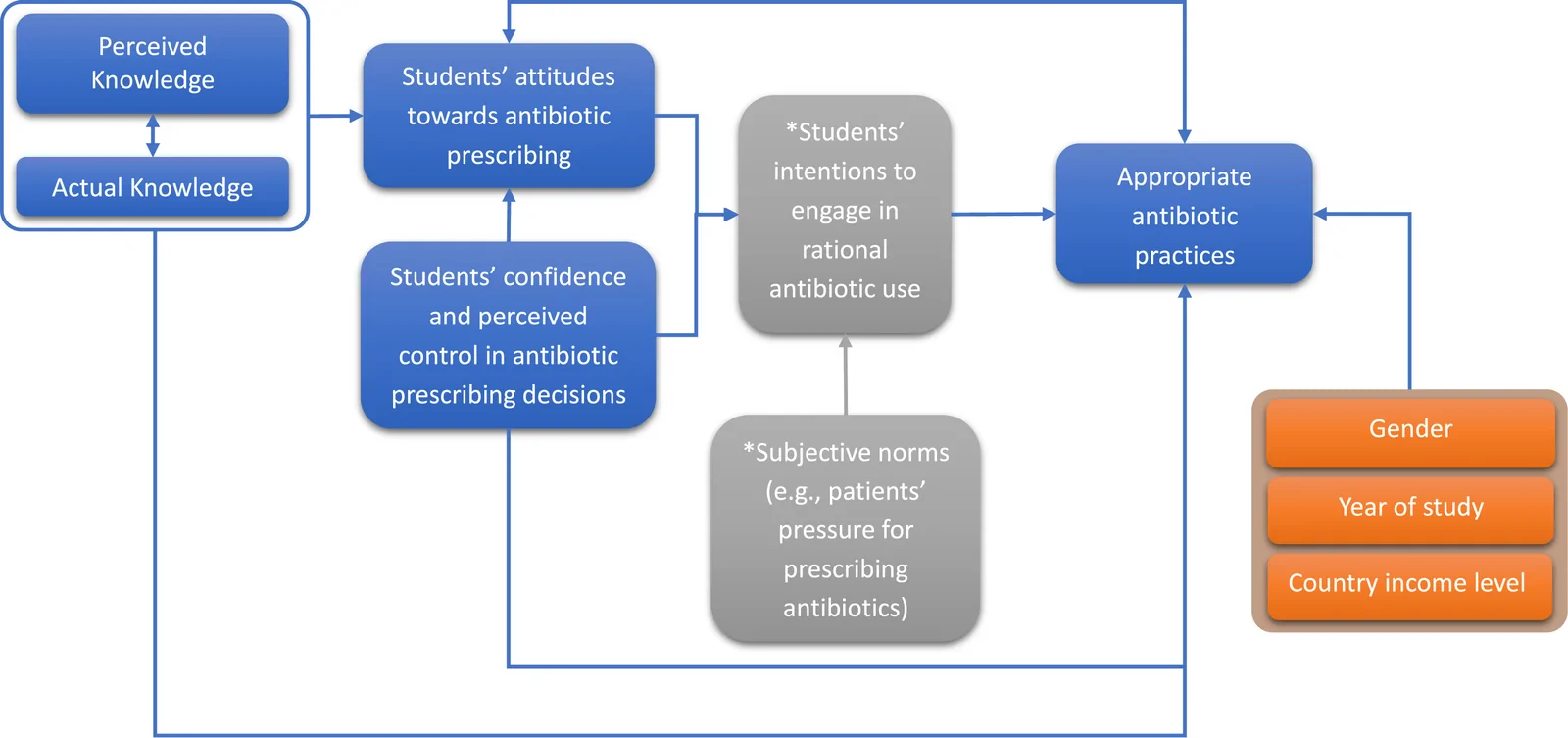
Adapting the theory of planned behaviour to the context of this study (* was not assessed in this study). This figure presents a hypothesis-generating conceptual model based on the TPB framework. It is intended to facilitate interpretation of the observed associations rather than to represent causal relationships established by this study.

Although confidence and attitudes were highly correlated, attitudes were found to carry more weight in determining prescribing practices among dental students in our study population. This can be explained through the theory of planned behaviour (TPB).^45^ Based on the TPB, the possibility of dental students engaging in appropriate antibiotic prescribing can be correlated with the strength of their intentions towards this issue. Attitudes towards antibiotic prescribing directly influence dental students’ intentions to engage in rational antibiotic use.^45^^,^^67^ While confidence overlaps with the perceived behavioural control (ie, the perceived difficulty level of the behaviour and its obstacles), without positive attitudes toward appropriate antibiotic use and prescription, confidence can even lead to the improper practice of overprescribing. In conclusion, our results suggest that attitudes may carry more significance than confidence for dental students, which aligns with Ajzen’s (1991) planned behaviour theory (Figure 2).^45^

In turn, attitude in this study was predicted by actual and perceived knowledge, confidence, and practice. Similar results were observed in previous studies, in which the actual knowledge of a surveyed Chinese and Indonesian public was associated with attitudes towards antibiotic use.^68^^,^^69^ The same applies to perceived knowledge, as a study in Hungary reported that pharmacists who are aware of the gaps in their knowledge might possess an appropriate attitude towards proper antibiotic use.^70^ This study also showed that attitude was predicted by confidence. A scoping review reported that medical students may hold misconceptions and overconfidence, which could be associated with improper antibiotic use.^71^ Furthermore, practice was also a predictor of the attitude towards antibiotic prescription. Opposite to what the TPB stated (that attitude, subjective norms, and perceived behavioural control with behaviour are mediated by intentions), practices can be the reason for reinforcing a positive attitude or changing a negative one. This suggests a reciprocal association between the components of the TPB, rather than going in one linear direction. Sussman and Gifford^72^ highlighted the possible reverse-causal relationship between intentions, attitudes, perceived control, and norms. Not only this, but the reciprocity is also reported between behaviour and attitudes.^73^ Literature showed that prior experiences can influence subsequent beliefs.^73^ In other words, a past behaviour has the potential of shaping future attitudes towards that same behavior.^74^ In our study, proper antibiotic prescription is associated with better attitudes. This means that if the health professionals were supported to have better practices, they would eventually develop better attitudes towards antibiotic use that can last for their entire career (Figure 2).

Interns demonstrated worse practice compared with students, which is consistent with previous studies. In one study in South Africa, intern medical doctors showed insufficient knowledge to achieve antibiotic stewardship. The interns themselves perceived that they lacked the preparedness for certain areas in prescribing antibiotics due to a lack of enough training.^75^ In India, a study reported that dental interns needed more education to have the necessary knowledge and preparedness to appropriately prescribe antibiotics in various clinical conditions.^76^ In Saudi Arabia, interns’ knowledge about antibiotic stewardship was almost similar to that of dental students, in which both groups’ knowledge was lower compared to other groups with more experience, such as general practitioners, postgraduates, and specialists.^77^ In another study in Serbia, postgraduate students had better practices compared to undergraduate ones; however, both under and postgraduate students showed a lack of proper knowledge of dental-related ethics, which might be a reflection of inappropriate antibiotic-prescribing practices.^78^

### Limitations

This study comes with several limitations. First, this study employed a cross-sectional design, following a convenience sampling method, which limits the generalizability of the results within the ten Arab countries. Although we were able to recruit a large number of dental students covering ten Arab nations, the sample size varied across countries, which also limits the representativeness of this sample to the dental students’ population in the included Arab nations. Furthermore, the study design limits the ability to infer causality between the exposure variables and antibiotic prescribing practices, which calls for future research to address this issue by conducting interventions or randomized trials, for instance. Second, social desirability and recall biases might be introduced, given that the responses on the survey items, such as confidence and practice, were self-reported and were not objectively assessed. Third, the survey did not capture some of the contextual factors that might be related to the antibiotic prescribing practices, such as sources of information, seeking peers’ or superiors’ advice or consultation before decision-making, or even patient-pressure (pushing to receive an antibiotic regimen).

Despite the aforementioned limitations, this exploratory study’s strengths are shown in its large sample size that covers a wide geographical area, validated scales, and a comprehensive statistical analysis approach. This study provides a strong foundation and a baseline for future research to be conducted addressing this issue among dental students and other dental practitioners, through educational interventions aiming to improve antibiotic prescribing practices in the region.

## Conclusion

The results of this study helped shed light on antibiotic prescribing practices and on understanding their determinants among dental students from ten Arab countries. Appropriate antibiotic prescribing was not only associated with better actual knowledge, but it was also associated with how students perceived their knowledge and their attitudes. Actual knowledge was positively associated with appropriate prescribing; however, better perceived knowledge was a stronger predictor of appropriate prescribing behaviours and practices. While their contribution to the total variance is small, positive attitudes still contributed significantly to the overall model, and they can represent one of the associated factors with appropriate prescribing practices.

Based on this study, it is recommended to prioritize improving perceived knowledge and access to guidance given their unique contribution to the total variance in prescribing practices. This is as critical as gaining a factual understanding of the rational use of antibiotics. However, shaping and nurturing dental students’ positive attitudes towards responsible antibiotic stewardship remains necessary as part of efforts to improve students’ practices. The dental schools in the Arab region need to adopt curricula that ensure students gain a comprehensive understanding of antibiotic stewardship and develop greater self-awareness of their ethical responsibility in prescribing. Audit-based strategies combined with education, feedback, and behaviour change messages tailored to each dentist can be adopted to improve their prescribing practices. Furthermore, governments, represented by the Ministry of Health, and other professional associations should collaborate with dental schools to deliver sustainable, effective continuing education programs, such as workshops and seminars, to keep students and new graduates up to date on best practices in antibiotic use. The continuing education programs need to include discussions of dentists’, especially young ones’, own practices to evaluate how appropriate their prescribing behaviour is and provide them feedback to overcome any misalignment with the recommended guidelines. Ultimately, dental schools and other organizations should collaborate to develop regional and context-specific antibiotic stewardship guidelines for the dental profession, grounded in recent research conducted both in the region and worldwide and taking into consideration the variation in access to dental care, antibiotic availability, and resistance rates.

## Conflict of interest

None disclosed.

## Appendix Supplementary materials

Image, application 1
