# Supplementary material for: Knowledge, Attitudes, and Practices of Antibiotic Prescribing Among Arab Dental Students: A Cross-Sectional Study
Source: Int Dent J. 2026 Sep 4;76(5):109785. doi: 10.1016/j.identj.2026.109785 (PMC13572304; doi:10.1016/j.identj.2026.109785)
Supplement: Supplementary file 1 [file mmc1.docx]

# Supplement 1:

**Questions used in this study**

| **Section** | **Item** | **Response Options** |
| --- | --- | --- |
| **Sociodemographic variables** | 1. Gender | 1. Male 2. Female |
|  | 2. University |  |
|  | 3. Year of Study | 1. 4th 2. 5th 3. Interns |
|  | 4. Do you have a family member/relative working in the medical field | 1. Yes 2. No |
| **Actual Knowledge** | 1. Antibiotics are effective against viruses | 1. True 2. False 3. Unsure |
|  | 2. Antibiotics are effective against cold infections | 1. True 2. False 3. Unsure |
|  | 3. Unnecessary use of antibiotics makes them ineffective | 1. True 2. False 3. Unsure |
|  | 4. Taking antibiotics has associated side effects or risks such as diarrhea, colitis, and allergies | 1. True 2. False 3. Unsure |
|  | 5. Every person treated with antibiotics is at an increased risk of antibiotic-resistant infection | 1. True 2. False 3. Unsure |
|  | 6. Antibiotic-resistant bacteria can spread from person to person | 1. True 2. False 3. Unsure |
|  | 7. Healthy people can carry antibiotic-resistant bacteria | 1. True 2. False 3. Unsure |
| **Perceived Knowledge** | 1. I know what antibiotic resistance is | 1. Strongly disagree 2. Disagree 3. Undecided 4. Agree Strongly 5. Agree 6. I do not understand the question 7. Not Applicable |
|  | 2. I have sufficient knowledge about how to use antibiotics appropriately for my current training | 1. Strongly disagree 2. Disagree 3. Undecided 4. Agree Strongly 5. Agree 6. I do not understand the question 7. Not Applicable |
|  | 3. I know what information to give to individuals about prudent use of antibiotics and antibiotic resistance | 1. Strongly disagree 2. Disagree 3. Undecided 4. Agree Strongly 5. Agree 6. I do not understand the question 7. Not Applicable |
| **Confidence** | 1. I am confident in making antibiotic prescribing decisions | 1. Strongly disagree 2. Disagree 3. Undecided 4. Agree 5. Strongly Agree 6. I do not understand the question 7. Not Applicable |
|  | 2. I have confidence in the antibiotic guidelines available to me | 1. Strongly disagree 2. Disagree 3. Undecided 4. Agree 5. Strongly Agree 6. I do not understand the question 7. Not Applicable |
| **Opportunity to Prescribe Antibiotics** | 1. I have easy access to the guidelines I need for managing infections | 1. Strongly disagree 2. Disagree 3. Undecided 4. Agree 5. Strongly Agree 6. I do not understand the question 7. Not Applicable |
|  | 2. I have easy access to the materials I need to advise on prudent antibiotic use and antibiotic resistance | 1. Strongly disagree 2. Disagree 3. Undecided 4. Agree 5. Strongly Agree 6. I do not understand the question 7. Not Applicable |
|  | 3. I have good opportunities to provide advice on prudent antibiotic use to individuals | 1. Strongly disagree 2. Disagree 3. Undecided 4. Agree 5. Strongly Agree 6. I do not understand the question 7. Not Applicable |
| **Good Practice** | 4. How often did you give out resources on prudent antibiotic use or management of infections to individuals during the last week? | 1. Once a day 2. More than once a day 3. Once a week 4. More than once a week 5. Rarely 6. Never 7. I do not remember 8. Not Applicable |
|  | 5. How often did you give out advice related to prudent antibiotic use or management of infections to an individual during the last week? | 1. Once a day 2. More than once a day 3. Once a week 4. More than once a week 5. Rarely 6. Never 7. I do not remember 8. Not Applicable |
| **Environmental & Animal health factors** | 1. Excessive use of antibiotics in livestock and food production is important in contributing to antibiotic resistance in bacteria in humans | 1. Strongly disagree 2. Disagree 3. Undecided 4. Agree 5. Strongly Agree 6. I do not understand the question 7. Not Applicable |
|  | 2. Environmental factors, such as wastewater in the environment, are important in contributing to antibiotic resistance in bacteria from humans | 1. Strongly disagree 2. Disagree 3. Undecided 4. Agree 5. Strongly Agree 6. I do not understand the question 7. Not Applicable |
| **Attitude to our role in Fighting Antibiotic Resistance** | 1. I have a key role in helping control antibiotic resistance | 1. Strongly disagree 2. Disagree 3. Undecided 4. Agree 5. Strongly Agree 6. I do not understand the question 7. Not Applicable |
|  | 2. I know there is a connection between my prescribing of antibiotics and the emergence and spread of antibiotic-resistant bacteria | 1. Strongly disagree 2. Disagree 3. Undecided 4. Agree 5. Strongly Agree 6. I do not understand the question 7. Not Applicable |
| **Bad Practices** | 1. How often would you have preferred not to prescribe an antibiotic but were not able to during the last week? | 1. Once a day 2. More than once a day 3. Once a week 4. More than once a week 5. Rarely 6. Never 7. I do not remember 8. Not Applicable |
|  | 2. How often did the fear of patient deterioration or the fear of complications lead you to prescribe antibiotics during the last week? | 1. Once a day 2. More than once a day 3. Once a week 4. More than once a week 5. Rarely 6. Never 7. I do not remember 8. Not Applicable |
|  | 3. How often did you prescribe antibiotics because it took less time than to explain the reason why they are not indicated during the last week? | 1. Once a day 2. More than once a day 3. Once a week 4. More than once a week 5. Rarely 6. Never 7. I do not remember 8. Not Applicable |
|  | 4. How often did you prescribe antibiotics in situations in which you can't conduct a follow-up of the patient during the last week? | 1. Once a day 2. More than once a day 3. Once a week 4. More than once a week 5. Rarely 6. Never 7. I do not remember 8. Not Applicable |
|  | 5. How often did you prescribe an antibiotic because you were uncertain about the diagnosis of infection during the last one week? | 1. Once a day 2. More than once a day 3. Once a week 4. More than once a week 5. Rarely 6. Never 7. I do not remember 8. Not Applicable |
|  | 6. How often did you prescribe an antibiotic to maintain the relationship with the patient during the last week? | 1. Once a day 2. More than once a day 3. Once a week 4. More than once a week 5. Rarely 6. Never 7. I do not remember 8. Not Applicable |
|  | 7. How often did you stop an antibiotic prescription earlier than the prescribed course length during the last week? | 1. Once a day 2. More than once a day 3. Once a week 4. More than once a week 5. Rarely 6. Never 7. I do not remember 8. Not Applicable |
|  | 8. How often did you prescribe a shorter course of treatment as compared to available guidelines during the last week? | 1. Once a day 2. More than once a day 3. Once a week 4. More than once a week 5. Rarely 6. Never 7. I do not remember 8. Not Applicable |
|  | 9. How often did you discontinue early (within three days after initiation) a treatment because bacterial infection was not likely after all during the last one week? | 1. Once a day 2. More than once a day 3. Once a week 4. More than once a week 5. Rarely 6. Never 7. I do not remember 8. Not Applicable |

# Supplement 2:

**Demographic characteristics**

| **Variable / Category** | **Egypt**  **N (%)** | **Iraq**  **N (%)** | **Jordan**  **N (%)** | **Kuwait**  **N (%)** | **Lebanon**  **N (%)** | **Libya**  **N (%)** | **Palestine**  **N (%)** | **Saudi Arabia**  **N (%)** | **Syria**  **N (%)** | **Tunisia**  **N (%)** | **Total**  **N (%)** |
| --- | --- | --- | --- | --- | --- | --- | --- | --- | --- | --- | --- |
| **Sex** | | | | | | | | | | | |
| **Female** | 234 (55.4) | 344 (60.5) | 103 (71.0) | 17 (63.0) | 49 (59.0) | 34 (75.6) | 375 (63.3) | 77 (42.3) | 40 (49.4) | 18 (81.8) | 1291 (59.5) |
| **Male** | 188 (44.6) | 225 (39.5) | 42 (29.0) | 10 (37.0) | 34 (41.0) | 11 (24.4) | 217 (36.7) | 105 (57.7) | 41 (50.6) | 4 (18.2) | 877 (40.5) |
| **Year of study** | | | | | | | | | | | |
| **4th Year** | 53 (12.6) | 257 (45.2) | 91 (62.8) | 4 (14.8) | 50 (60.2) | 18 (40.0) | 216 (36.5) | 33 (18.1) | 18 (22.2) | 1  (4.5) | 741 (34.2) |
| **5th Year** | 193 (45.7) | 267 (46.9) | 21 (14.5) | 10 (37.0) | 28 (33.7) | 7 (15.6) | 190 (32.1) | 39 (21.4) | 26 (32.1) | 5 (22.7) | 786 (36.3) |
| **Interns** | 176 (41.7) | 45 (7.9) | 33 (22.8) | 13 (48.1) | 5  (6.0) | 20 (44.4) | 186 (31.4) | 110 (60.4) | 37 (45.7) | 16 (72.7) | 641 (29.6) |
| **Do you have a family member/relative working in the medical field?** | | | | | | | | | | | |
| **No** | 149 (35.3) | 238 (41.8) | 50 (34.5) | 6 (22.2) | 45 (54.2) | 21 (46.7) | 240 (40.5) | 72 (39.6) | 29 (35.8) | 11 (50.0) | 861 (39.7) |
| **Yes** | 273 (64.7) | 331 (58.2) | 95 (65.5) | 21 (77.8) | 38 (45.8) | 24 (53.3) | 352 (59.5) | 110 (60.4) | 52 (64.2) | 11 (50.0) | 1307 (60.3) |
| **Total N** | 422 | 569 | 145 | 27 | 83 | 45 | 592 | 182 | 81 | 22 | 2168 |
